# The Flaws and Human Harms of Animal Experimentation

**DOI:** 10.1017/S0963180115000079

**Published:** 2015-10

**Authors:** AYSHA AKHTAR

**Keywords:** animal research, medical testing, human health, human ethics, drug development, animal ethics

## Abstract

Nonhuman animal (“animal”) experimentation is typically defended by arguments that it is reliable, that animals provide sufficiently good models of human biology and diseases to yield relevant information, and that, consequently, its use provides major human health benefits. I demonstrate that a growing body of scientific literature critically assessing the validity of animal experimentation generally (and animal modeling specifically) raises important concerns about its reliability and predictive value for human outcomes and for understanding human physiology. The unreliability of animal experimentation across a wide range of areas undermines scientific arguments in favor of the practice. Additionally, I show how animal experimentation often significantly harms humans through misleading safety studies, potential abandonment of effective therapeutics, and direction of resources away from more effective testing methods. The resulting evidence suggests that the collective harms and costs to humans from animal experimentation outweigh potential benefits and that resources would be better invested in developing human-based testing methods.

## Introduction

Annually, more than 115 million animals are used worldwide in experimentation or to supply the biomedical industry.^1^ Nonhuman animal (hereafter “animal”) experimentation falls under two categories: basic (i.e., investigation of basic biology and human disease) and applied (i.e., drug research and development and toxicity and safety testing). Regardless of its categorization, animal experimentation is intended to inform human biology and health sciences and to promote the safety and efficacy of potential treatments. Despite its use of immense resources, the animal suffering involved, and its impact on human health, the question of animal experimentation’s efficacy has been subjected to little systematic scrutiny.^2^

Although it is widely accepted that medicine should be *evidence based*, animal experimentation as a means of informing human health has generally not been held, in practice, to this standard. This fact makes it surprising that animal experimentation is typically viewed as the default and gold standard of preclinical testing and is generally supported without critical examination of its validity. A survey published in 2008 of anecdotal cases and statements given in support of animal experimentation demonstrates how it has not and could not be validated as a necessary step in biomedical research, and the survey casts doubt on its predictive value.^3^ I show that animal experimentation is poorly predictive of human outcomes,^4^ that it is unreliable across a wide category of disease areas,^5^ and that existing literature demonstrates the unreliability of animal experimentation, thereby undermining scientific arguments in its favor. I further show that the collective harms that result from an unreliable practice tip the ethical scale of harms and benefits against continuation in much, if not all, of experimentation involving animals.^6^

## Problems of Successful Translation to Humans of Data from Animal Experimentation

Although the unreliability and limitations of animal experimentation have increasingly been acknowledged, there remains a general confidence within much of the biomedical community that they can be overcome.^7^ However, three major conditions undermine this confidence and explain why animal experimentation, regardless of the disease category studied, fails to reliably inform human health: (1) the effects of the laboratory environment and other variables on study outcomes, (2) disparities between animal models of disease and human diseases, and (3) species differences in physiology and genetics. I argue for the critical importance of each of these conditions.

### The Influence of Laboratory Procedures and Environments on Experimental Results

Laboratory procedures and conditions exert influences on animals’ physiology and behaviors that are difficult to control and that can ultimately impact research outcomes. Animals in laboratories are involuntarily placed in artificial environments, usually in windowless rooms, for the duration of their lives. Captivity and the common features of biomedical laboratories—such as artificial lighting, human-produced noises, and restricted housing environments—can prevent species-typical behaviors, causing distress and abnormal behaviors among animals.^8^ Among the types of laboratory-generated distress is the phenomenon of contagious anxiety.^9^ Cortisone levels rise in monkeys watching other monkeys being restrained for blood collection.^10^ Blood pressure and heart rates elevate in rats watching other rats being decapitated.^11^ Routine laboratory procedures, such as catching an animal and removing him or her from the cage, in addition to the experimental procedures, cause significant and prolonged elevations in animals’ stress markers.^12^ These stress-related changes in physiological parameters caused by the laboratory procedures and environments can have significant effects on test results.^13^ Stressed rats, for example, develop chronic inflammatory conditions and intestinal leakage, which add variables that can confound data.^14^

A variety of conditions in the laboratory cause changes in neurochemistry, genetic expression, and nerve regeneration.^15^ In one study, for example, mice were genetically altered to develop aortic defects. Yet, when the mice were housed in larger cages, those defects almost completely disappeared.^16^ Providing further examples, typical noise levels in laboratories can damage blood vessels in animals, and even the type of flooring on which animals are tested in spinal cord injury experiments can affect whether a drug shows a benefit.^17^

In order to control for potential confounders, some investigators have called for standardization of laboratory settings and procedures.^18^ One notable effort was made by Crabbe et al. in their investigation of the potential confounding influences of the laboratory environment on six mouse behaviors that are commonly studied in neurobehavioral experiments. Despite their “extraordinary lengths to equate test apparatus, testing protocols, and all possible features of animal husbandry” across three laboratories, there were systematic differences in test results in these labs.^19^ Additionally, different mouse strains varied markedly in all behavioral tests, and for some tests the magnitude of genetic differences depended on the specific testing laboratory. The results suggest that there are important influences of environmental conditions and procedures specific to individual laboratories that can be difficult—perhaps even impossible—to eliminate. These influences can confound research results and impede extrapolation to humans.

### The Discordance between Human Diseases and Animal Models of Diseases

The lack of sufficient congruence between animal models and human diseases is another significant obstacle to translational reliability. Human diseases are typically artificially induced in animals, but the enormous difficulty of reproducing anything approaching the complexity of human diseases in animal models limits their usefulness.^20^ Even if the design and conduct of an animal experiment are sound and standardized, the translation of its results to the clinic may fail because of disparities between the animal experimental model and the human condition.^21^

Stroke research presents one salient example of the difficulties in modeling human diseases in animals. Stroke is relatively well understood in its underlying pathology. Yet accurately modeling the disease in animals has proven to be an exercise in futility. To address the inability to replicate human stroke in animals, many assert the need to use more standardized animal study design protocols. This includes the use of animals who represent both genders and wide age ranges, who have comorbidities and preexisting conditions that occur naturally in humans, and who are consequently given medications that are indicated for human patients.^22^ In fact, a set of guidelines, named STAIR, was implemented by a stroke roundtable in 1999 (and updated in 2009) to standardize protocols, limit the discrepancies, and improve the applicability of animal stroke experiments to humans.^23^ One of the most promising stroke treatments later to emerge was NXY-059, which proved effective in animal experiments. However, the drug failed in clinical trials, despite the fact that the set of animal experiments on this drug was considered the poster child for the new experimental standards.^24^ Despite such vigorous efforts, the development of STAIR and other criteria has yet to make a recognizable impact in clinical translation.^25^

Under closer scrutiny, it is not difficult to surmise why animal stroke experiments fail to successfully translate to humans even with new guidelines. Standard stroke medications will likely affect different species differently. There is little evidence to suggest that a female rat, dog, or monkey sufficiently reproduces the physiology of a human female. Perhaps most importantly, reproducing the preexisting conditions of stroke in animals proves just as difficult as reproducing stroke pathology and outcomes. For example, most animals don’t naturally develop significant atherosclerosis, a leading contributor to ischemic stroke. In order to reproduce the effects of atherosclerosis in animals, researchers clamp their blood vessels or artificially insert blood clots. These interventions, however, do not replicate the elaborate pathology of atherosclerosis and its underlying causes. Reproducing human diseases in animals requires reproducing the *predisposing* diseases, also a formidable challenge. The inability to reproduce the disease in animals so that it is congruent in relevant respects with human stroke has contributed to a high failure rate in drug development. More than 114 potential therapies initially tested in animals failed in human trials.^26^

Further examples of repeated failures based on animal models include drug development in cancer, amyotrophic lateral sclerosis (ALS), traumatic brain injury (TBI), Alzheimer’s disease (AD), and inflammatory conditions. Animal cancer models in which tumors are artificially induced have been the basic translational model used to study key physiological and biochemical properties in cancer onset and propagation and to evaluate novel treatments. Nevertheless, significant limitations exist in the models’ ability to faithfully mirror the complex process of human carcinogenesis.^27^ These limitations are evidenced by the high (among the highest of any disease category) clinical failure rate of cancer drugs.^28^ Analyses of common mice ALS models demonstrate significant differences from human ALS.^29^ The inability of animal ALS models to predict beneficial effects in humans with ALS is recognized.^30^ More than twenty drugs have failed in clinical trials, and the only U.S. Food and Drug Administration (FDA)–approved drug to treat ALS is Riluzole, which shows notably marginal benefit on patient survival.^31^ Animal models have also been unable to reproduce the complexities of human TBI.^32^ In 2010, Maas et al. reported on 27 large Phase 3 clinical trials and 6 unpublished trials in TBI that all failed to show human benefit after showing benefit in animals.^33^ Additionally, even after success in animals, around 172 and 150 drug development failures have been identified in the treatment of human AD^34^ and inflammatory diseases,^35^ respectively.

The high clinical failure rate in drug development across all disease categories is based, at least in part, on the inability to adequately model human diseases in animals and the poor predictability of animal models.^36^ A notable systematic review, published in 2007, compared animal experimentation results with clinical trial findings across interventions aimed at the treatment of head injury, respiratory distress syndrome, osteoporosis, stroke, and hemorrhage.^37^ The study found that the human and animal results were in accordance only half of the time. In other words, the animal experiments were no more likely than a flip of the coin to predict whether those interventions would benefit humans.

In 2004, the FDA estimated that 92 percent of drugs that pass preclinical tests, including “pivotal” animal tests, fail to proceed to the market.^38^ More recent analysis suggests that, despite efforts to improve the predictability of animal testing, the failure rate has actually increased and is now closer to 96 percent.^39^ The main causes of failure are lack of effectiveness and safety problems that were not predicted by animal tests.^40^

Usually, when an animal model is found wanting, various reasons are proffered to explain what went wrong—poor methodology, publication bias, lack of preexisting disease and medications, wrong gender or age, and so on. These factors certainly require consideration, and recognition of each potential difference between the animal model and the human disease motivates renewed efforts to eliminate these differences. As a result, scientific progress is sometimes made by such efforts. However, the high failure rate in drug testing and development, despite attempts to improve animal testing, suggests that these efforts remain insufficient to overcome the obstacles to successful translation that are inherent to the use of animals. Too often ignored is the well-substantiated idea that these models are, for reasons summarized here, intrinsically lacking in relevance to, and thus highly unlikely to yield useful information about, human diseases.^41^

### Interspecies Differences in Physiology and Genetics

Ultimately, even if considerable congruence were shown between an animal model and its corresponding human disease, interspecies differences in physiology, behavior, pharmacokinetics, and genetics would significantly limit the reliability of animal studies, even after a substantial investment to improve such studies. In spinal cord injury, for example, drug testing results vary according to which species and even which strain within a species is used, because of numerous interspecies and interstrain differences in neurophysiology, anatomy, and behavior.^42^ The micropathology of spinal cord injury, injury repair mechanisms, and recovery from injury varies greatly among different strains of rats and mice. A systematic review found that even among the most standardized and methodologically superior animal experiments, testing results assessing the effectiveness of methylprednisolone for spinal cord injury treatment varied considerably among species.^43^ This suggests that factors inherent to the use of animals account for some of the major differences in results.

Even rats from the same strain but purchased from different suppliers produce different test results.^44^ In one study, responses to 12 different behavioral measures of pain sensitivity, which are important markers of spinal cord injury, varied among 11 strains of mice, with no clear-cut patterns that allowed prediction of how each strain would respond.^45^ These differences influenced how the animals responded to the injury and to experimental therapies. A drug might be shown to help one strain of mice recover but not another. Despite decades of using animal models, not a single neuroprotective agent that ameliorated spinal cord injury in animal tests has proven efficacious in clinical trials to date.^46^

Further exemplifying the importance of physiological differences among species, a 2013 study reported that the mouse models used extensively to study human inflammatory diseases (in sepsis, burns, infection, and trauma) have been misleading. The study found that mice differ greatly from humans in their responses to inflammatory conditions. Mice differed from humans in what genes were turned on and off and in the timing and duration of gene expression. The mouse models even differed from one another in their responses. The investigators concluded that “our study supports higher priority to focus on the more complex human conditions rather than relying on mouse models to study human inflammatory disease.”^47^ The different genetic responses between mice and humans are likely responsible, at least in part, for the high drug failure rate. The authors stated that every one of almost 150 clinical trials that tested candidate agents’ ability to block inflammatory responses in critically ill patients failed.

Wide differences have also become apparent in the regulation of the same genes, a point that is readily seen when observing differences between human and mouse livers.^48^ Consistent phenotypes (observable physical or biochemical characteristics) are rarely obtained by modification of the same gene, even among different strains of mice.^49^ Gene regulation can substantially differ among species and may be as important as the presence or absence of a specific gene. Despite the high degree of genome conservation, there are critical differences in the order and function of genes among species. To use an analogy: as pianos have the same keys, humans and other animals share (largely) the same genes. Where we mostly differ is in the way the genes or keys are expressed. For example, if we play the keys in a certain order, we hear Chopin; in a different order, we hear Ray Charles; and in yet a different order, it’s Jerry Lee Lewis. In other words, the same keys or genes are expressed, but their different orders result in markedly different outcomes.

Recognizing the inherent genetic differences among species as a barrier to translation, researches have expressed considerable enthusiasm for genetically modified (GM) animals, including transgenic mice models, wherein human genes are inserted into the mouse genome. However, if a human gene is expressed in mice, it will likely function differently from the way it functions in humans, being affected by physiological mechanisms that are unique in mice. For example, a crucial protein that controls blood sugar in humans is missing in mice.^50^ When the human gene that makes this protein was expressed in genetically altered mice, it had the opposite effect from that in humans: it caused *loss* of blood sugar control in mice. Use of GM mice has failed to successfully model human diseases and to translate into clinical benefit across many disease categories.^51^ Perhaps the primary reason why GM animals are unlikely to be much more successful than other animal models in translational medicine is the fact that the “humanized” or altered genes are still in nonhuman animals.

In many instances, nonhuman primates (NHPs) are used instead of mice or other animals, with the expectation that NHPs will better mimic human results. However, there have been sufficient failures in translation to undermine this optimism. For example, NHP models have failed to reproduce key features of Parkinson’s disease, both in function and in pathology.^52^ Several therapies that appeared promising in both NHPs and rat models of Parkinson’s disease showed disappointing results in humans.^53^ The campaign to prescribe hormone replacement therapy (HRT) in millions of women to prevent cardiovascular disease was based in large part on experiments on NHPs. HRT is now known to *increase* the risk of these diseases in women.^54^

HIV/AIDS vaccine research using NHPs represents one of the most notable failures in animal experimentation translation. Immense resources and decades of time have been devoted to creating NHP (including chimpanzee) models of HIV. Yet all of about 90 HIV vaccines that succeeded in animals failed in humans.^55^ After HIV vaccine gp120 failed in clinical trials, despite positive outcomes in chimpanzees, a *BMJ* article commented that important differences between NHPs and humans with HIV misled researchers, taking them down unproductive experimental paths.^56^ Gp120 failed to neutralize HIV grown and tested in cell culture. However, because the serum protected chimpanzees from HIV infection, two Phase 3 clinical trials were undertaken^57^—a clear example of how expectations that NHP data are more predictive than data from other (in this case, cell culture) testing methods are unproductive and harmful. Despite the repeated failures, NHPs (though not chimpanzees or other great apes) remain widely used for HIV research.

The implicit assumption that NHP (and indeed any animal) data are reliable has also led to significant and unjustifiable human suffering. For example, clinical trial volunteers for gp120 were placed at unnecessary risk of harm because of unfounded confidence in NHP experiments. Two landmark studies involving thousands of menopausal women being treated with HRT were terminated early because of increased stroke and breast cancer risk.^58^ In 2003, Elan Pharmaceuticals was forced to prematurely terminate a Phase 2 clinical trial when an investigational AD vaccine was found to cause brain swelling in human subjects. No significant adverse effects were detected in GM mice or NHPs.^59^

In another example of human suffering resulting from animal experimentation, six human volunteers were injected with an immunomodulatory drug, TGN 1412, in 2006.^60^ Within minutes of receiving the experimental drug, all volunteers suffered a severe adverse reaction resulting from a life-threatening cytokine storm that led to catastrophic systemic organ failure. The compound was designed to dampen the immune system, but it had the *opposite* effect in humans. Prior to this first human trial, TGN 1412 was tested in mice, rabbits, rats, and NHPs with no ill effects. NHPs also underwent repeat-dose toxicity studies and were given 500 times the human dose for at least four consecutive weeks.^61^ None of the NHPs manifested the ill effects that humans showed almost immediately after receiving minute amounts of the test drug. Cynomolgus and rhesus monkeys were specifically chosen because their CD28 receptors demonstrated similar affinity to TGN 1412 as human CD28 receptors. Based on such data as these, it was confidently concluded that results obtained from these NHPs would most reliably predict drug responses in humans—a conclusion that proved devastatingly wrong.

As exemplified by the study of HIV/AIDS, TGN 1412, and other experiences,^62^ experiments with NHPs are not necessarily any more predictive of human responses than experiments with other animals. The repeated failures in translation from studies with NHPs belie arguments favoring use of *any* nonhuman species to study human physiology and diseases and to test potential treatments. If experimentation using chimpanzees and other NHPs, our closest genetic cousins, are unreliable, how can we expect research using other animals to be reliable? The bottom line is that animal experiments, no matter the species used or the type of disease research undertaken, are highly unreliable—and they have too little predictive value to justify the resultant risks of harms for humans, for reasons I now explain.

## The Collective Harms That Result from Misleading Animal Experiments

As medical research has explored the complexities and subtle nuances of biological systems, problems have arisen because the *differences* among species along these subtler biological dimensions far outweigh the *similarities*, as a growing body of evidence attests. These profoundly important—and often undetected—differences are likely one of the main reasons human clinical trials fail.^63^

“Appreciation of differences” and “caution” about extrapolating results from animals to humans are now almost universally recommended. But, in practice, how does one take into account differences in drug metabolism, genetics, expression of diseases, anatomy, influences of laboratory environments, and species- and strain-specific physiologic mechanisms—and, in view of these differences, discern what is applicable to humans and what is not? If we cannot determine which physiological mechanisms in which species and strains of species are applicable to humans (even setting aside the complicating factors of different caging systems and types of flooring), the usefulness of the experiments must be questioned.

It has been argued that some information obtained from animal experiments is better than no information.^64^ This thesis neglects how misleading information can be worse than no information from animal tests. The use of nonpredictive animal experiments can cause human suffering in at least two ways: (1) by producing misleading safety and efficacy data and (2) by causing potential abandonment of useful medical treatments and misdirecting resources away from more effective testing methods.

Humans are harmed because of misleading animal testing results. Imprecise results from animal experiments may result in clinical trials of biologically faulty or even harmful substances, thereby exposing patients to unnecessary risk and wasting scarce research resources.^65^ Animal toxicity studies are poor predictors of toxic effects of drugs in humans.^66^ As seen in some of the preceding examples (in particular, stroke, HRT, and TGN1412), humans have been significantly harmed because investigators were misled by the safety and efficacy profile of a new drug based on animal experiments.^67^ Clinical trial volunteers are thus provided with raised hopes and a false sense of security because of a misguided confidence in efficacy and safety testing using animals.

An equal if indirect source of human suffering is the opportunity cost of abandoning promising drugs because of misleading animal tests.^68^ As candidate drugs generally proceed down the development pipeline and to human testing based largely on successful results in animals^69^ (i.e., positive efficacy and negative adverse effects), drugs are sometimes not further developed due to unsuccessful results in animals (i.e., negative efficacy and/or positive adverse effects). Because much pharmaceutical company preclinical data are proprietary and thus publicly unavailable, it is difficult to know the number of missed opportunities due to misleading animal experiments. However, of every 5,000–10,000 potential drugs investigated, only about 5 proceed to Phase 1 clinical trials.^70^ Potential therapeutics may be abandoned because of results in animal tests that do not apply to humans.^71^ Treatments that fail to work or show some adverse effect in animals because of species-specific influences may be abandoned in preclinical testing even if they may have proved effective and safe in humans if allowed to continue through the drug development pipeline.

An editorial in *Nature Reviews Drug Discovery* describes cases involving two drugs in which animal test results from species-specific influences could have derailed their development. In particular, it describes how tamoxifen, one of the most effective drugs for certain types of breast cancer, “would most certainly have been withdrawn from the pipeline” if its propensity to cause liver tumor in rats had been discovered in preclinical testing rather than after the drug had been on the market for years.^72^ Gleevec provides another example of effective drugs that could have been abandoned based on misleading animal tests: this drug, which is used to treat chronic myelogenous leukemia (CML), showed serious adverse effects in at least five species tested, including severe liver damage in dogs. However, liver toxicity was not detected in human cell assays, and clinical trials proceeded, which confirmed the absence of significant liver toxicity in humans.^73^ Fortunately for CML patients, Gleevec is a success story of predictive human-based testing. Many useful drugs that have safely been used by humans for decades, such as aspirin and penicillin, may not have been available today if the current animal testing regulatory requirements were in practice during their development.^74^

A further example of near-missed opportunities is provided by experiments on animals that delayed the acceptance of cyclosporine, a drug widely and successfully used to treat autoimmune disorders and prevent organ transplant rejection.^75^ Its immunosuppressive effects differed so markedly among species that researchers judged that the animal results limited any direct inferences that could be made to humans. Providing further examples, PharmaInformatic released a report describing how several blockbuster drugs, including aripiprazole (Abilify) and esomeprazole (Nexium), showed low oral bioavailability in animals. They would likely not be available on the market today if animal tests were solely relied on. Understanding the implications of its findings for drug development in general, PharmaInformatic asked, “Which other blockbuster drugs would be on the market today, if animal trials would have not been used to preselect compounds and drug-candidates for further development?”^76^ These near-missed opportunities and the overall 96 percent failure rate in clinical drug testing strongly suggest the unsoundness of animal testing as a precondition of human clinical trials and provide powerful evidence for the need for a new, human-based paradigm in medical research and drug development.

In addition to potentially causing abandonment of useful treatments, use of an invalid animal disease model can lead researchers and the industry in the wrong research direction, wasting time and significant investment.^77^ Repeatedly, researchers have been lured down the wrong line of investigation because of information gleaned from animal experiments that later proved to be inaccurate, irrelevant, or discordant with human biology. Some claim that we do not know which benefits animal experiments, particularly in basic research, may provide down the road. Yet human lives remain in the balance, waiting for effective therapies. Funding must be strategically invested in the research areas that offer the most promise.

The opportunity costs of continuing to fund unreliable animal tests may impede development of more accurate testing methods. Human organs grown in the lab, human organs on a chip, cognitive computing technologies, 3D printing of human living tissues, and the Human Toxome Project are examples of new human-based technologies that are garnering widespread enthusiasm. The benefit of using these testing methods in the preclinical setting over animal experiments is that they are based on *human* biology. Thus their use eliminates much of the guesswork required when attempting to extrapolate physiological data from other species to humans. Additionally, these tests offer whole-systems biology, in contrast to traditional in vitro techniques. Although they are gaining momentum, these human-based tests are still in their relative infancy, and funding must be prioritized for their further development. The recent advancements made in the development of more predictive, human-based systems and biological approaches in chemical toxicological testing are an example of how newer and improved tests have been developed because of a shift in prioritization.^78^ Apart from toxicology, though, financial investment in the development of human-based technologies generally falls far short of investment in animal experimentation.^79^

## Conclusion

The unreliability of applying animal experimental results to human biology and diseases is increasingly recognized. Animals are in many respects biologically and psychologically similar to humans, perhaps most notably in the shared characteristics of pain, fear, and suffering.^80^ In contrast, evidence demonstrates that critically important physiological and genetic differences between humans and other animals can invalidate the use of animals to study human diseases, treatments, pharmaceuticals, and the like. In significant measure, animal models specifically, and animal experimentation generally, are inadequate bases for predicting clinical outcomes in human beings in the great bulk of biomedical science. As a result, humans can be subject to significant and avoidable harm.

The data showing the unreliability of animal experimentation and the resultant harms to humans (and nonhumans) undermine long-standing claims that animal experimentation is necessary to enhance human health and therefore ethically justified. Rather, they demonstrate that animal experimentation poses significant costs and harms to human beings. It is possible—as I have argued elsewhere—that animal research is more costly and harmful, on the whole, than it is beneficial to human health.^81^ When considering the ethical justifiability of animal experiments, we should ask if it is ethically acceptable to deprive humans of resources, opportunity, hope, and even their lives by seeking answers in what may be the wrong place. In my view, it would be better to direct resources away from animal experimentation and into developing more accurate, human-based technologies.

## Notes

1. TaylorK, GordonN, LangleyG, HigginsW. Estimates for worldwide laboratory animal use in 2005. Alternatives to Laboratory Animals 2008;36:327–42.1866209610.1177/026119290803600310

2. Systematic reviews that have been conducted generally reveal the unreliability and poor predictability of animal tests. See Perel P, Roberts I, Sena E, Wheble P, Briscoe C, Sandercock P, et al. Comparison of treatment effects between animal experiments and clinical trials: Systematic review. *BMJ* 2007;334:197. See also Pound P, Bracken MB. Is animal research sufficiently evidence based to be a cornerstone of biomedical research? *BMJ* 2014;348:g3387. See Godlee F. How predictive and productive is animal research? *BMJ* 2014;348:g3719. See BenatarM. Lost in translation: Treatment trials in the SOD 1 mouse and in human ALS. Neurobiology Disease 2007;26:1–1310.1016/j.nbd.2006.12.01517300945. And see AkhtarAZ, PippinJJ, SanduskyCB. Animal studies in spinal cord injury: A systematic review of methylprednisolone. Alternatives to Laboratory Animals 2009;37:43–62.1929257510.1177/026119290903700108

3. MathewsRAJ. Medical progress depends on animal models—doesn’t it? Journal of the Royal Society of Medicine 2008;101:95–8.1829963110.1258/jrsm.2007.070164PMC2254450

4. See ShanksN, GreekR, GreekJ. Are animal models predictive for humans? Philosophy, Ethics, and Humanities in Medicine 2009;4:210.1186/1747-5341-4-2PMC264286019146696. See also WallRJ, ShaniM. Are animal models as good as we think? Theriogenology 2008;69:2–9.1798872510.1016/j.theriogenology.2007.09.030

5. See note 3, Mathews 2008. See also HartungT, ZurloJ. Food for thought… alternative approaches for medical countermeasures to biological and chemical terrorism and warfare. ALTEX 2012;29:251–602284725310.14573/altex.2012.3.251. See LeistM, HartungT. Inflammatory findings on species extrapolations: Humans are definitely no 70-kg mice. Archives in Toxicology 2013;87:563–710.1007/s00204-013-1038-0PMC360459623503654. See MakIWY, EvaniewN, GhertM. Lost in translation: Animal models and clinical trials in cancer treatment. American Journal in Translational Research 2014;6:114–18PMC390222124489990. And see PippinJ. Animal research in medical sciences: Seeking a convergence of science, medicine, and animal law. South Texas Law Review 2013;54:469–511.

6. For an overview of the harms-versus-benefits argument, see LaFolletteH. Animal experimentation in biomedical research In: BeauchampTL, FreyRG, eds. The Oxford Handbook of Animal Ethics. Oxford: Oxford University Press; 2011:812–18.

7. See JuckerM. The benefits and limitations of animal models for translational research in neurodegenerative diseases. Nature Medicine 2010;16:1210–1410.1038/nm.222421052075. See Institute of Medicine. *Improving the Utility and Translation of Animal Models for Nervous System Disorders: Workshop Summary.* Washington, DC: The National Academies Press; 2013. And see DegryseAL, LawsonWE. Progress towards improving animal models for IPF. American Journal of Medical Science 2011;341:444–9.10.1097/MAJ.0b013e31821aa000PMC310307821613932

8. See MorganKN, TromborgCT. Sources of stress in captivity. Applied Animal Behaviour Science 2007;102:262–302. See HartPC, BergnerCL, DufourBD, SmolinskyAN, EganRJ, LaPorteL, et al. Analysis of abnormal repetitive behaviors in experimental animal models In WarrickJE, KauleffAV, eds. Translational Neuroscience and Its Advancement of Animal Research Ethics. New York: Nova Science; 2009:71–82. See LutzC, WellA, NovakM. Stereotypic and self-injurious behavior in rhesus macaques: A survey and retrospective analysis of environment and early experience. American Journal of Primatology 2003;60:1–151276693810.1002/ajp.10075. And see BalcombeJP, BarnardND, SanduskyC. Laboratory routines cause animal stress. Contemporary Topics in Laboratory Animal Science 2004;43:42–51.15669134

9. Suckow MA, Weisbroth SH, Franklin CL. *The Laboratory Rat*. 2nd ed. Burlington, MA: Elsevier Academic Press; 2006, at 323.

10. Flow BL, Jaques JT. Effect of room arrangement and blood sample collection sequence on serum thyroid hormone and cortisol concentrations in cynomolgus macaques (*Macaca fascicularis*). *Contemporary Topics in Laboratory Animal Science* 1997;36:65–8.

11. See note 8, Balcombe et al. 2004.

12. See note 8, Balcombe et al. 2004.

13. Baldwin A, Bekoff M. Too stressed to work. *New Scientist* 2007;194:24.

14. See note 13, Baldwin, Bekoff 2007.

15. AkhtarA, PippinJJ, SanduskyCB. Animal models in spinal cord injury: A review. Reviews in the Neurosciences 2008;19:47–60.1856182010.1515/revneuro.2008.19.1.47

16. See note 13, Baldwin, Bekoff 2007.

17. See note 15, Akhtar et al. 2008.

18. See MacleodMR, O’CollinsT, HowellsDW, DonnanGA. Pooling of animal experimental data reveals influence of study design and publication bias. Stroke 2004;35:1203–81506032210.1161/01.STR.0000125719.25853.20. See also O’ Neil BJ, Kline JA, Burkhart K, Younger J. Research fundamentals: V. The use of laboratory animal models in research. *Academic Emergency Medicine* 1999;6:75–82.

19. CrabbeJC, WahlstenD, DudekBC. Genetics of mouse behavior: Interactions with laboratory environment. Science 1999;284:1670–2, at 1670.1035639710.1126/science.284.5420.1670

20. See Curry SH. Why have so many drugs with stellar results in laboratory stroke models failed in clinical trials? A theory based on allometric relationships. *Annals of the New York Academy of Sciences* 2003;993:69–74. See also DirnaglU. Bench to bedside: The quest for quality in experimental stroke research. Journal of Cerebral Blood Flow & Metabolism 2006;26:1465–781652541310.1038/sj.jcbfm.9600298.

21. van der WorpHB, HowellsDW, SenaES, PorittMJ, RewellS, O’CollinsV, et al. Can animal models of disease reliably inform human studies? PLoS Medicine 2010;7:e10002452036102010.1371/journal.pmed.1000245PMC2846855.

22. See note 20, Dirnagl 2006. See also SenaE, van der WorpB, HowellsD, MacleodM. How can we improve the pre-clinical development of drugs for stroke? Trends in Neurosciences 2007;30:433–9.1776533210.1016/j.tins.2007.06.009

23. See Gawrylewski A. The trouble with animal models: Why did human trials fail? *The Scientist* 2007;21:44. See also FisherM, FeuersteinG, HowellsDW, HurnPD, KentTA, SavitzSI, et al. Update of the stroke therapy academic industry roundtable preclinical recommendations. Stroke 2009;40:2244–50.1924669010.1161/STROKEAHA.108.541128PMC2888275

24. See note 23, Gawrylewski 2007. There is some dispute as to how vigorously investigators adhered to the suggested criteria. Nevertheless, NXY-059 animal studies were considered an example of preclinical studies that most faithfully adhered to the STAIR criteria. For further discussion see also WangMM, GuohuaX, KeepRF. Should the STAIR criteria be modified for preconditioning studies? Translational Stroke Research 2013;4:3–142345888510.1007/s12975-012-0219-0PMC3580874.

25. See note 24, Wang et al. 2013.

26. O’CollinsVE, MacleodMR, DonnanGA, HorkyLL, van der WorpBH, HowellsDW. 1,026 experimental treatments in acute stroke. Annals of Neurology 2006;59:467–71645331610.1002/ana.20741.

27. See note 5, Mak et al. 2014.

28. See note 5, Mak et al. 2014.

29. See PerrinS. Preclinical research: Make mouse studies work. Nature 2014;507:423–52467854010.1038/507423a. See also, generally, Wilkins HM, Bouchard RJ, Lorenzon NM, Linseman DA. Poor correlation between drug efficacies in the mutant SOD1 mouse mode versus clinical trials of ALS necessitates the development of novel animal models for sporadic motor neuron disease. In: Costa A, Villalba E, eds. *Horizons in Neuroscience Research.* Vol. 5. Hauppauge, NY: Nova Science; 2011:1–39.

30. TraynorBJ, BruijnL, ConwitR, BealF, O’NeillG, FaganSC, et al. Neuroprotective agents for clinical trials in ALS: A systematic assessment. Neurology 2006;67:20–71683207210.1212/01.wnl.0000223353.34006.54.

31. SinhaG. Another blow for ALS. Nature Biotechnology 2013;31:185. See also note 30, Traynor et al. 2006.

32. See MoralesDM, MarklundN, LeboldD, ThompsonHJ, PitkanenA, MaxwellWL, et al. Experimental models of traumatic brain injury: Do we really need a better mousetrap? Neuroscience 2005;136:971–891624284610.1016/j.neuroscience.2005.08.030. See also XiongYE, MahmoodA, ChoppM. Animal models of traumatic brain injury. Nature Reviews Neuroscience 2013;14:128–422332916010.1038/nrn3407PMC3951995. And see commentary by Farber: Farber N. Drug development in brain injury. *International Brain Injury Association*; available at http://www.internationalbrain.org/articles/drug-development-in-traumatic-brain-injury/ (last accessed 7 Dec 2014).

33. MaasAI, RoozenbeekB, ManleyGT. Clinical trials in traumatic brain injury: Past experience and current developments. Neurotherapeutics 2010;7:115–26.2012950310.1016/j.nurt.2009.10.022PMC5084118

34. SchneiderLS, MangialascheF, AndreasenN, FeldmanH, GiacobiniE, JonesR, et al. Clinical trials and late-stage drug development in Alzheimer’s disease: An appraisal from 1984 to 2014. Journal of Internal Medicine 2014;275:251–832460580810.1111/joim.12191PMC3956752.

35. SeokJ, WarrenHS, CuencaAG, MindrinosMN, BakerHV, XuW, et al. Genomic responses in mouse models poorly mimic human inflammatory diseases. Proceedings of the National Academy of Sciences USA 2013;110:3507–12.10.1073/pnas.1222878110PMC358722023401516

36. PalfreymanMG, CharlesV, BlanderJ. The importance of using human-based models in gene and drug discovery. Drug Discovery World 2002 Fall:33–40.

37. See note 2, Perel et al. 2007.

38. Harding A. More compounds failing phase I. *The Scientist* 2004 Sept 13; available at http://www.the-scientist.com/?articles.view/articleNo/23003/title/More-compounds-failing-Phase-I/ (last accessed 2 June 2014).

39. See note 5, Pippin 2013.

40. See note 5, Hartung, Zurlo 2012.

41. WiebersDO, AdamsHP, WhisnantJP. Animal models of stroke: Are they relevant to human disease? Stroke 1990;21:1–3.240554510.1161/01.str.21.1.1

42. See note 15, Akhtar et al. 2008.

43. See note 2, Akhtar et al. 2009.

44. LonjonN, PrietoM, HatonH, BrøchnerCB, BauchetL, CostalatV, et al. Minimum information about animal experiments: Supplier is also important. Journal of Neuroscience Research 2009;87:403–7.1879828210.1002/jnr.21871

45. MogilJS, WilsonSG, BonK, LeeSE, ChungK, RaberP, et al. Heritability of nociception I: Responses of 11 inbred mouse strains on 12 measures of nociception. Pain 1999;80:67–82.1020471910.1016/s0304-3959(98)00197-3

46. TatorH, HashimotoR, RaichA, NorvellD, FehlingMG, HarropJS, et al. Translational potential of preclinical trials of neuroprotection through pharmacotherapy for spinal cord injury. Journal of Neurosurgery: Spine 2012;17:157–229.2298538210.3171/2012.5.AOSPINE12116

47. See note 35, Seok et al. 2013, at 3507.

48. OdomDT, DowellRD, JacobsenES, GordonW, DanfordTW, MacIsaacKD, et al. Tissue-specific transcriptional regulation has diverged significantly between human and mouse. Nature Genetics 2007;39:730–21752997710.1038/ng2047PMC3797512.

49. HorrobinDF. Modern biomedical research: An internally self-consistent universe with little contact with medical reality? Nature Reviews Drug Discovery 2003;2:151–4.10.1038/nrd101212563306

50. VassilopoulousS, EskC, HoshinoS, FunkeBH, ChenCY, PlocikAM, et al. A role for the CHC22 clathrin heavy-chain isoform in human glucose metabolism. Science 2009;324:1192–6.1947818210.1126/science.1171529PMC2975026

51. See Guttman-YasskyE, KruegerJG. Psoriasis: Evolution of pathogenic concepts and new therapies through phases of translational research. British Journal of Dermatology 2007;157:1103–151771456010.1111/j.1365-2133.2007.08135.x. See also The mouse model: Less than perfect, still invaluable. *Johns Hopkins Medicine*; available at http://www.hopkinsmedicine.org/institute_basic_biomedical_sciences/news_events/articles_and_stories/model_organisms/201010_mouse_model.html (last accessed 10 Dec 2014). See note 23, Gawrylewski 2007. See note 2, Benatar 2007. See note 29, Perrin 2014 and Wilkins et al. 2011. See Cavanaugh S, Pippin J, Barnard N. Animal models of Alzheimer disease: Historical pitfalls and a path forward. *ALTEX* online first; 2014 Apr 10. And see WoodroofeA, ColemanRA. ServiceNote: Human tissue research for drug discovery. Genetic Engineering and Biotechnology News 2007;27:18.

52. LaneE, DunnettS. Animal models of Parkinson’s disease and L-dopa induced dyskinesia: How close are we to the clinic? Psychopharmacology 2008;199:303–12.1789902010.1007/s00213-007-0931-8

53. See note 52, Lane, Dunnett 2008.

54. See note 5, Pippin 2013.

55. BaileyJ. An assessment of the role of chimpanzees in AIDS vaccine research. Alternatives to Laboratory Animals 2008;36:381–428.1882633110.1177/026119290803600403

56. TonksA. The quest for the AIDs vaccine. BMJ 2007;334:1346–81760002010.1136/bmj.39240.416968.ADPMC1906671.

57. JohnstonMI, FauciAS. An HIV vaccine—evolving concepts. New England Journal of Medicine 2007;356:2073–81.1750770610.1056/NEJMra066267

58. See RossouwJE, AndersenGL, PrenticeRL, LaCroixAZ, KooperberfC, StefanickML, et al. Risks and benefits of estrogen plus progestin in healthy menopausal women: Principle results from the Women’s Health Initiative randomized controlled trial. JAMA 2002;288:321–331211739710.1001/jama.288.3.321. See also AndersenGL, LimacherA, AssafAR, BassfordT, BeresfordSA, BlackH, et al. Effects of conjugated equine estrogen in postmenopausal women with hysterectomy: The Women’s Health Initiative randomized controlled trial. JAMA 2004;291:1701–121508269710.1001/jama.291.14.1701.

59. LemereCA. Developing novel immunogens for a safe and effective Alzheimer’s disease vaccine. Progress in Brain Research 2009;175:831966065010.1016/S0079-6123(09)17506-4PMC2814339.

60. Allen A. Of mice and men: The problems with animal testing. *Slate* 2006 June 1; available at http://www.slate.com/articles/health_and_science/medical_examiner/2006/06/of_mice_or_men.html (last accessed 10 Dec 2014).

61. AttarwalaH. TGN1412: From discovery to disaster. Journal of Young Pharmacists 2010;2:332–62104249610.4103/0975-1483.66810PMC2964774.

62. See HoganRJ. Are nonhuman primates good models for SARS? PLoS Medicine 2006;3:1656–710.1371/journal.pmed.0030411PMC157633517002511. See also BaileyJ. Non-human primates in medical research and drug development: A critical review. Biogenic Amines 2005;19:235–55.

63. See note 4, Wall, Shani 2008.

64. LemonR, DunnettSB. Surveying the literature from animal experiments: Critical reviews may be helpful—not systematic ones. BMJ 2005;330:977–8.1586080210.1136/bmj.330.7498.977PMC557132

65. RobertsI, KwanI, EvansP, HaigS. Does animal experimentation inform human health care? Observations from a systematic review of international animal experiments on fluid resuscitation. BMJ 2002;324:474–61185905310.1136/bmj.324.7335.474PMC1122396.

66. See note 60,Allen 2006. See also HeywoodR. Target organ toxicity. Toxicology Letters 1981;8:349–58730296510.1016/0378-4274(81)90125-9. See FletcherAP. Drug safety tests and subsequent clinical experience. Journal of the Royal Society of Medicine 1978;71:693–6.71275010.1177/014107687807100915PMC1436259

67. See note 60, Allen 2006. See note 5, Pippin 2013. See also GreekR, GreekJ. Animal research and human disease. JAMA 2000;283:743–410683047.

68. See note 60, Allen 2006. See also note 5, Leist, Hartung 2013.

69. Food and Drug Administration. Development & approval process (drugs); available at http://www.fda.gov/Drugs/DevelopmentApprovalProcess/ (last accessed 7 Dec 2014). See also http://www.fda.gov/drugs/resourcesforyou/consumers/ucm143534.htm (last accessed 7 Dec 2014).

70. Drug discovery pipeline. IRSF; available at http://www.rettsyndrome.org/research-programs/programmatic-overview/drug-discovery-pipeline (last accessed 24 Sept 2014).

71. See note 60, Allen 2006.

72. Follow the yellow brick road. *Nature Reviews Drug Discovery* 2003;2:167, at 167.

73. See note 5, Pippin 2013.

74. For data on aspirin, see HartungT. Per aspirin as astra … Alternatives to Laboratory Animals 2009;37(Suppl 2):45–72010501110.1177/026119290903702S10. See also note 5, Pippin 2013. For data on penicillin, see KoppanyiT, AveryMA. Species differences and the clinical trial of new drugs: A review. Clinical Pharmacology and Therapeutics 1966;7:250–70532717910.1002/cpt196672250. See also SchneiersonSS, PerlmanE. Toxicity of penicillin for the Syrian hamster. Proceedings of the Society for Experimental Biology and Medicine 1956;91:229–30.1329776110.3181/00379727-91-22221

75. See note 67, Greek, Greek 2000.

76. Oral bioavailability of blockbuster drugs in humans and animals. *PharmaInformatic*. available at http://www.pharmainformatic.com/html/blockbuster_drugs.html (last accessed 19 Sept 2014).

77. Sams-DoddF. Strategies to optimize the validity of disease models in the drug discovery process. Drug Discovery Today 2006;11:355–631658097810.1016/j.drudis.2006.02.005.

78. ZurloJ. No animals harmed: Toward a paradigm shift in toxicity testing. Hastings Center Report 2012;42 Suppl:s23–610.1002/hast.10423138426.

79. There is no direct analysis of the amount of money spent on animal testing versus alternatives across all categories; however, in 2008 the *Chronicle of Higher Education* reported that funding of research involving animals (under basic research) of the National Institute of Health (NIH) remained steady at about 42 percent since 1990. See Monastersky R. Protesters fail to slow animal research. *Chronicle of Higher Education* 2008:54. In 2012, NIH director Francis Collins noted that the NIH’s support for basic research has held steady at 54 percent of the agency’s budget for decades. The remainder of the NIH’s budget is heavily funded toward clinical research, suggesting that *preclinical* human-based testing methods are much less funded. See also Wadman M. NIH director grilled over translational research centre. *Nature News Blog* 2012 Mar 20. Available at http://blogs.nature.com/news/2012/03/nih-director-grilled-over-translational-research-center.html (last accessed 5 Mar 2015). There is no data that suggests that the NIH’s funding of animal experimentation has decreased. A 2010 analysis estimates that at least 50 percent of the NIH’s extramural funding is directed into animal research; see GreekR, GreekJ. Is the use of sentient animals in basic research justifiable? Philosophy, Ethics, and Humanities in Medicine 2010;5:1410.1186/1747-5341-5-14PMC294961920825676.

80. For a helpful discussion on animal pain, fear, and suffering, see DeGraziaD. Taking Animals Seriously: Mental Lives and Moral Status. New York: Cambridge University Press; 1996:116–23.

81. See Akhtar A. *Animals and Public Health: Why Treating Animals Better Is Critical to Human Welfare*. Hampshire, UK: Palgrave Macmillan; 2012:chap. 5.

